# Mechanical Ventilation in COVID-19 Patients: a question from Age to Frailty

**DOI:** 10.14336/AD.2022.0819

**Published:** 2023-04-01

**Authors:** Antonio M Esquinas, Dipasri Bhattacharya, Mohanchandra Mandal

**Affiliations:** ^1^Intensive Care Unit, Hospital Morales, Meseguer, Murcia, Spain.; ^2^Department of Anaesthesiology, Pain Medicine, and Critical Care, R. G. Kar Medical College, Kolkata, West Bengal, India.; ^3^Department of Anaesthesiology and Critical Care, Institute of Post Graduate Medical Education and Research, Kolkata, India.

## To Editor,

We have read with great interest the study of Ecarnot F, et al. [1] where the authors have found that incidence of intubation and mechanical ventilation is more in younger patients compared to older patients with severe COVID-19 infection. The patient with heart rate more than 110/minute and requiring CPAP during admission are more likely to receive mechanical ventilation. Although the authors have done a nice work to find out the risk factors among COVID-19 patients requiring mechanical ventilation we consider that there are certain points that need further clarification.

Firstly, the study finds that younger patients who finally needed intubation and mechanical ventilation had greater oxygen requirement at admission. It would be interesting to know the details of patient data such as Sat O2 and PaO_2_/FiO_2_ ratio. The parameters such as SatO2 < 94%, PaO_2_/FiO_2_ < 300 and PaO2 < 60 was found to bear two- to three-fold correlation with adverse outcome [2].

Secondly, authors mentioned that the patients who needed CPAP during admission more likely needed mechanical ventilation. For people receiving CPAP, the ROX index is a strong predictor of adverse outcome, as well as mortality [3]. The ROX index, PaO_2_/FiO_2_ ratio and high respiratory rate are important predictors of severity [4]. ROX index before the start of CPAP and within 24 hours of initiation can predict responsiveness to NIV [3].

Thirdly, Frailty is a better predictor of outcome of severity in Covid 19 patients than chronological age. Frailty has increased vulnerability to external stressors resulting in decreased functional reserve [5].

Ecarnot F, et al. [1] observed a median frailty index (FI) score 0.088 (0.03, 0.20) which indicates that only a minority of their cohort (n=1344) was actually frail. There is a general agreement that a FI score of 0.25 is the cut-off above which a patient is considered to be frail [6]. It should be appreciated that assessment of FI score would help the clinicians and intensivists in identifying the patients at risk of adverse outcomes.
